# The admixture maximum likelihood test to test for association between rare variants and disease phenotypes

**DOI:** 10.1186/1471-2105-14-177

**Published:** 2013-06-06

**Authors:** Jonathan P Tyrer, Qi Guo, Douglas F Easton, Paul DP Pharoah

**Affiliations:** 1 Centre for Cancer Genetic Epidemiology, Department of Oncology, University of Cambridge, Cambridge, UK; 2 Centre for Cancer Genetic Epidemiology, Department of Public Health and Primary Care, University of Cambridge, Cambridge, UK

## Abstract

**Background:**

The development of genotyping arrays containing hundreds of thousands of rare variants across the genome and advances in high-throughput sequencing technologies have made feasible empirical genetic association studies to search for rare disease susceptibility alleles. As single variant testing is underpowered to detect associations, the development of statistical methods to combine analysis across variants – so-called “burden tests” - is an area of active research interest. We previously developed a method, the admixture maximum likelihood test, to test multiple, common variants for association with a trait of interest. We have extended this method, called the rare admixture maximum likelihood test (RAML), for the analysis of rare variants. In this paper we compare the performance of RAML with six other burden tests designed to test for association of rare variants.

**Results:**

We used simulation testing over a range of scenarios to test the power of RAML compared to the other rare variant association testing methods. These scenarios modelled differences in effect variability, the average direction of effect and the proportion of associated variants. We evaluated the power for all the different scenarios. RAML tended to have the greatest power for most scenarios where the proportion of associated variants was small, whereas SKAT-O performed a little better for the scenarios with a higher proportion of associated variants.

**Conclusions:**

The RAML method makes no assumptions about the proportion of variants that are associated with the phenotype of interest or the magnitude and direction of their effect. The method is flexible and can be applied to both dichotomous and quantitative traits and allows for the inclusion of covariates in the underlying regression model. The RAML method performed well compared to the other methods over a wide range of scenarios. Generally power was moderate in most of the scenarios, underlying the need for large sample sizes in any form of association testing.

## Background

Genome-wide association studies (GWAS) have successfully identified common genetic variants, mainly common single nucleotide polymorphisms (SNP), with small to modest effects for many complex human diseases and traits [1]. However, for most diseases common susceptibility variants identified to date explain only a small proportion of the heritable component of disease risk. A range of genetic models may explain the missing heritability from a very large number of common variants that confer risks too small to be detected to many rare variants with stronger effects. Rare disease-susceptibility alleles identified so far have mostly been in the coding sequence of genes and are associated with higher disease risks than known common susceptibility alleles.

Until recently a limited understanding of the architecture of rare genetic variation across the genome and limitations of genotyping technologies have restricted the search for rare disease-susceptibility alleles to the analysis of a small number of candidate genes for specific diseases. The development of genotyping arrays containing hundreds of thousands of rare variants across the genome and advances in high-throughput sequencing technologies have made feasible empirical genetic association studies to search for rare disease susceptibility alleles. Even so, standard methods used for association testing, in which association with the trait of interest is tested one variant at a time, are limited by statistical power. As a result there has been an increase in interest in alternative analytic methods in which the information across multiple variant sites is combined – for example all variants in a specified gene or genomic region. Depending on the underlying model of genetic association these “burden” tests can enhance statistical power. Examples of these include the cohort allelic sum test (CAST) [2,3], the combined multi-variate and collapsing (CMC) test [2], the weighted sum test (WST) [4], the variable-threshold test (VTT) [5], the C-alpha test (CAT) [6], the sequence kernel association test (SKAT) [7,8], and the estimated regression coefficient (EREC) test [9].

The CAST and CMC methods collapse information on all rare variants within a region into a single dichotomous variable for each subject and then apply a univariate test. Rare variants are defined by a fixed threshold for minor allele frequency. The WST is a non-parametric test in which rare variants are grouped according to function (e.g. gene) and each individual is scored by a sum of the mutation counts weighted by the variance under the null hypothesis. The VT approach selects an allele frequency threshold by maximising the test statistic over all thresholds and assesses statistical significance by permutation. The major limitation of these simple burden tests is that they do not account for the direction of effects of the functional alleles that are assumed to be the same. However, a gene harbouring phenotypically relevant variation could include a handful of rare Mendelian mutations that cause disease, some variants that moderately increase or decrease risk, along with numerous variants of no effect.

The CAT contrasts the variance of each observed count with the expected variance. However, the method does not allow for covariate adjustment. SKAT is a score-based variance-component test that makes no assumption about directionality of effect by performing multiple regressions of a phenotype on genotype for all variants in a region [7]. P-values are calculated analytically. The most recent implementation, called SKAT-O is a generalisation of the C-alpha test that enables the incorporation of covariates and is more powerful than simple burden tests over a range of plausible genetic models [8]. The EREC test is a modification of the WST and VTT in which the weighting is based on the estimated regression coefficient.

We have previously developed an admixture maximum likelihood (AML) test to test for association of multiple common genetic variants with the trait of interest [10]. The method is flexible and can be applied to both disease and quantitative traits and can include covariates. In this paper we propose an extension of the AML test, hereafter called RAML (rare admixture maximum likelihood test), for the analysis of uncommon variants. RAML takes account of variants that increase or decrease risk or have no effect on risk. We have compared the performance of RAML with SKAT-O and with five tests implemented by Score-SEQ (two fixed threshold methods with the minor allele frequency threshold set to 1 percent and 5 percent, a modified WST, a modified VTT and the EREC test) [9].

## Methods

The RAML method provides an omnibus test for joint effects of multiple variants on a phenotype and formulates the alternative hypothesis in terms of the probability that a given variant is associated with disease (α), the average effect of the associated variants (η) and the expected standard error of this effect (σ). The effect of each variant is estimated as the signed z-statistic (Z) from the score test. To generate the omnibus test statistic the distribution of the effects under the alternative hypothesis need to be defined. It is desirable to use a distribution with a conjugate prior so that the likelihood will have a tractable computational form. We defined this as a normal distribution of the z-statistic. Using the z-statistic gives flexibility to incorporate covariates and the approach can be easily extended to quantitative trait and survival-time analyses. The average expected z-statistic can be positive or negative corresponding to an excess of deleterious or protective variants respectively. Given that a variant is associated with the phenotype the likelihood of observing the test statistic (Z)

(1) $$\int p\left(T|\mu =x\right)p\left(\mu =x|\eta ,\sigma \right)\mathrm{dx}$$

To solve this equation we can think of it in terms of the sum of two normal distributions *x ~ N*(0,1) and *y ~ N*(μ,σ^2^). The likelihood of observing *S = x + y* can then be expressed as

(2) $$\int p\left(S|y=Y\right)p\left(y=Y|N\left(\mu ,\sigma^{2}\right)\right)\mathrm{dy}$$

The distribution of *Z* given μ is distributed as a *N*(0,1) distribution so it can be seen that equations (1) and (2) are equivalent. Thus in the case that a variant is associated the test statistic Z is distributed as *N*(η,1 + σ^2^). The log-likelihood is of the form

(3) $$l\left(\alpha ,\eta ,\sigma \right)=\sum_{i}\text{log}\left(1-\alpha +\alpha L_{1i}/L_{0i}\right)$$

where *L*_*1i*_ is the likelihood given the variant is associated (which will be from a *N*(η,1 + σ^2^) distribution) and *L*_*0i*_ is the null likelihood (which is from a *N*(0,1) distribution). Thus the log-likelihood can be expressed as

(4) $$l\left(\alpha ,\eta ,\sigma \right)=\sum_{i}\text{log}\left(1-\alpha +\frac{\alpha }{\sqrt{1+\sigma^{2}}}\text{exp}\left\{-\frac{1}{2}\left(\frac{{\left(x_{i}-\eta \right)}^{2}}{1+\sigma^{2}}-x_{i}{}^{2}\right)\right\}\right)$$

which we maximised using the Bound Optimization BY Quadratic Approximation (BOBYQA) algorithm [11].

One major problem in testing multiple variants in the same region of the genome is correlation due to linkage disequilibrium (LD), although this may be less of an issue for rare genetic variation. A permutation test can account for type I error, but there is still a loss of power as variants in strong LD with each other have a disproportionate effect on the test statistic. To deal with this, the RAML groups variants using a single link cluster approach, such that every variant in a group has a squared correlation (r^2^) greater than a specified threshold with at least one other variant in the same group. For each group a proxy variant is generated, where the proxy is the maximum number of rare alleles for any variant in the group (0, 1 or 2) carried by each subject. The default r^2^ threshold is set to 0.9. This deals with perfectly correlated variants whilst still being able to test most variants individually.

Restricting the parameter space to plausible values should help to improve power. Therefore bounds for α, η and σ^2^ need to be defined as well as a definition of a rare variant. We set the bound for specifying a variant as rare as having a minor allele frequency of 0.04 or less. We expect most variants will not be associated so we set an upper limit for α of 0.2. As we want to be able to model both strong protective and deleterious effects we took the bounds for η to be from −5 to 5. We chose the minimum value of σ^2^ to be 0.25. This represents the minimum amount of variability we could expect about the associated variant effects. Different choices of bounds will do better in some scenarios and worse in others. Our aim was to try to find a reasonable choice for likely effects that are seen in genetic association studies.

Simulation testing

We simulated population data using phased haplotypes from the 1000 Genome Project data (http://www.1000genomes.org/). In order to determine the risk associated with each haplotype the risk associated with each variant was based on seven different scenarios with risk distributions defined below

1. *β* ~ *N*(2*σ*, *σ*^2^)

2. 80 per cent of risk variants *β* ~ *N*(2*σ*, *σ*^2^), 20 per cent of risk variants *β* ~ *N*(−2*σ*, *σ*^2^)

3. *β* ~ *N*(*σ*, *σ*^2^)

4. 80 per cent of risk variants *β* ~ *N*(*σ*, *σ*^2^), 20 per cent of risk variants *β* ~ *N*(−*σ*, *σ*^2^)

5. *β* ~ *N*(*σ*/2, *σ*^2^)

6. 80 per cent of risk variants *β* ~ *N*(*σ*/2, *σ*^2^), 20 per cent of risk variants *β* ~ *N*(−*σ*/2, *σ*^2^)

7. *β* ~ *N*(0, *σ*^2^)

These scenarios are similar to the ones presented by Lee *et al.*[8] with the main difference being that we added some variability. The parameters were chosen to give roughly the same power for each scenario.

Two haplotypes were selected at random for each individual. The overall risk associated with any pair of haplotypes was calculated under a log-additive model by summing the risks from each causal variant carried. An individual in the population was assigned as a case or control at random based on this risk and a disease prevalence of 10 per cent. Two thousand cases and two thousand controls were then selected randomly from the population.

For each risk distribution we tested three scenarios in which 5%, 10% or 20% of variants were causal. The effect was set as proportional to the log of the variant minor allele frequency (p). The standard error (σ) varied under the different distributions: For 5% of associated variants,

$$\sigma =-0.04\text{log}\left(p\right)k$$

where *k* is 1 for distributions 1 and 2, 1.5 for distributions 3 and 4, 2 for distribution 5 and 6 and 2.5 for distribution 7; for 10% and 20% of associated variants the average effect was 0.7 and 0.5 times this respectively.

We sampled haplotypes from three different regions of the genome (TERT chr5: 1253287–1295162, BRCA2 chr13: 32889617–32973809, BRCA1 chr17: 41243452–41277500) in order to evaluate the influence of different local LD structure on the different tests. Two hundred replicate data sets were simulated under each of the 21 different scenarios. We derived the power of each test as the proportion of replicates for which the empirical significance level achieved P < 0.05, P < 0.01 and P < 0.001.

We compared the RAML method to SKAT-O using the default weights and the five methods included in the program ScoreSeq. We also applied our tests to three different genes to evaluate the effects that varying genomic architecture has on the relative efficacy of the methods.

In order to evaluate the power for a larger sample size and slightly stronger effects we repeated the simulations for four thousand cases and four thousand controls across the same genomic regions with the effect of associated variants being 25 per cent larger. This data set was used to compare the performance of RAML with SKAT-O.

## Results and discussion

There were 145 rare variants (MAF < 0.04) in *BRCA1* (109 variants with MAF < 0.01, 27 with 0.01 < MAF < 0.02, 9 with 0.02 < MAF < 0.04), 274 rare variants in *BRCA2* (196, 22, 56) and 193 rare variants in *TERT* (155, 23, 15). The power of the seven methods for 21 scenarios at the three thresholds for statistical significance are shown in Tables 1, 2 and 3 for *BRCA1*, *BRCA2* and *TERT* respectively. Generally, power was limited (< 50 per cent) for all methods across a wide range of plausible scenarios for the underlying genetic model. The RAML test had the greatest power for most scenarios for both *BRCA2* and *TERT*. For *BRCA1*, RAML tended to have the greatest power for the scenarios with a small proportion of associated variants, whereas SKAT-O performed a little better for the scenarios with a higher proportion of associated variants. Whether 100% or 80% of variants are associated with effects in the same direction did not change the relative efficacy of the two methods.

**Table 1:** Power (%) of seven methods to detect association of rare variants under seven scenarios for underlying genetic architecture using data simulated for BRCA1 in 2000 cases and 2000 controls

		**Threshold for significance**																				
		**P < 0.001**							**P < 0.01**							**P < 0.05**						
Scenario*	**Proportion of variants associated**	**RAML**	**SKAT-O**	**T1**	**T5**	**WST**	**VTT**	**EREC**	**RAML**	**SKAT-O**	**T1**	**T5**	**WST**	**VTT**	**EREC**	**RAML**	**SKAT-O**	**T1**	**T5**	**WST**	**VTT**	**EREC**
1	0.05	**5.0**	4.5	3.0	2.5	2.0	3.0	3.0	**15.0**	11.0	10.0	7.5	8.0	7.0	10.0	**30.0**	29.0	22.5	18.5	20.5	19.5	26.0
2	0.05	5.0	**6.5**	1.5	2.0	1.0	1.5	3.0	**11.5**	**11.5**	4.5	6.0	5.5	9.0	8.0	**28.0**	21.5	14.5	10.5	13.5	17.0	16.0
3	0.05	**7.5**	5.0	2.5	0.5	1.5	1.5	4.0	**13.5**	11.0	7.0	6.0	7.0	7.0	8.0	25.5	**26.0**	16.0	19.5	17.0	17.5	22.5
4	0.05	**7.0**	4.0	2.0	1.0	1.0	1.0	2.5	**14.0**	12.5	5.0	3.5	5.0	4.5	8.0	**26.0**	23.5	12.5	12.0	9.5	10.0	21.0
5	0.05	**6.5**	5.0	2.0	2.0	1.5	2.0	3.5	**14.5**	9.5	5.0	2.5	3.5	5.5	6.5	**27.5**	18.0	11.5	8.0	10.0	13.5	14.5
6	0.05	**9.0**	4.5	1.0	0.5	0.5	1.0	3.5	**16.0**	11.0	5.0	4.0	3.0	3.0	8.5	**26.0**	20.5	12.5	10.5	11.0	10.5	18.5
7	0.05	**8.5**	5.0	1.0	1.5	0.5	1.5	4.0	**16.0**	8.0	3.0	4.0	3.5	4.0	7.5	**24.5**	21.5	8.0	8.5	6.5	10.0	16.5
1	0.10	**7.5**	6.0	6.0	2.0	5.0	4.5	5.5	14.5	**16.0**	11.0	8.0	10.0	11.0	15.0	**30.0**	**30.0**	24.5	21.5	20.0	20.5	25.0
2	0.10	2.5	**3.5**	1.5	2.0	1.5	0.5	1.5	8.5	8.5	6.0	5.5	7.0	6.5	**10.5**	**26.0**	24.0	19.5	17.5	17.5	17.5	20.5
3	0.10	4.5	**5.0**	3.0	2.5	3.0	1.5	3.5	**10.5**	8.5	7.0	4.5	5.5	7.5	8.5	**22.0**	19.5	15.0	11.5	14.0	15.5	18.5
4	0.10	**3.5**	3.0	2.0	2.0	1.5	1.5	2.5	9.5	**10.5**	6.0	3.0	4.5	3.5	7.5	**22.0**	18.5	13.5	13.0	13.0	12.5	17.5
5	0.10	4.0	**4.5**	3.0	1.0	1.5	3.0	2.5	**10.5**	9.0	5.5	3.0	5.0	5.5	9.0	**23.0**	21.0	13.0	11.5	14.0	14.5	22.0
6	0.10	2.5	**3.5**	1.5	1.0	1.5	1.0	1.5	**9.0**	**9.0**	5.0	2.5	5.5	3.5	5.5	**21.0**	18.0	10.0	9.0	9.0	11.0	16.5
7	0.10	**4.0**	3.0	1.0	1.0	0.5	2.0	3.5	**11.0**	10.5	2.5	5.0	3.0	3.0	7.5	**25.5**	20.0	11.5	11.5	12.0	12.5	17.0
1	0.20	6.5	**7.5**	**7.5**	4.0	5.5	4.5	**7.5**	23.5	**27.5**	23.5	18.5	24.5	20.5	25.5	40.0	**44.5**	44.0	40.0	**44.5**	39.0	43.5
2	0.20	4.0	**6.5**	4.5	2.0	4.5	4.0	4.5	**14.0**	12.5	**14.0**	7.0	10.0	9.5	12.5	23.0	**26.5**	23.5	16.5	22.5	23.0	24.0
3	0.20	5.0	**6.0**	5.5	3.5	5.0	5.5	5.0	14.5	**17.0**	15.0	12.5	16.5	13.0	**17.0**	27.5	**36.5**	31.0	28.5	34.0	30.0	35.5
4	0.20	2.5	**3.0**	1.5	1.5	1.5	1.0	2.0	7.5	**10.5**	5.0	6.5	5.5	5.0	8.0	18.5	**21.5**	16.0	14.0	16.5	17.0	18.5
5	0.20	4.5	**5.0**	1.0	1.0	1.5	1.0	2.5	**10.5**	9.0	7.0	4.0	4.5	6.0	8.0	24.0	**25.5**	17.5	13.5	15.0	14.5	20.5
6	0.20	0.5	**1.0**	0.0	0.0	0.5	0.0	0.5	**6.5**	**6.5**	3.5	2.5	2.0	1.5	4.5	17.0	**20.0**	10.0	12.0	11.0	8.5	18.5
7	0.20	3.0	**3.5**	0.5	0.5	1.0	0.5	0.5	9.0	**9.5**	3.0	4.0	2.5	3.5	6.5	**23.5**	21.5	12.0	9.5	12.0	12.0	17.5

Method with greatest power emboldened.

* See text for description of genetic architecture for each scenario.

*RAML* Rare admixture maximum likelihood, *SKAT-O* sequence kernel association test, *T1*fixed threshold test 1 per cent MAF, *T5* fixed threshold test 5 per cent MAF, *WST* weighted sum test, *VTT* variable threshold test, *EREC* estimated regression coefficient test.

**Table 2:** Power (%) of seven methods to detect association of rare variants under seven scenarios for underlying genetic architecture using data simulated for BRCA2 in 2000 cases and 2000 controls

		**Threshold for significance**																				
		**P < 0.001**							**P < 0.01**							**P < 0.05**						
Scenario*	**Proportion of variants associated**	**RAML**	**SKAT-O**	**T1**	**T5**	**WST**	**VTT**	**EREC**	**RAML**	**SKAT-O**	**T1**	**T5**	**WST**	**VTT**	**EREC**	**RAML**	**SKAT-O**	**T1**	**T5**	**WST**	**VTT**	**EREC**
1	0.05	**19.5**	2.5	0.5	2.5	1.5	4.5	5.5	**41.0**	7.5	6.0	7.0	6.0	14.5	11.0	**57.5**	18.0	21.5	15.0	15.5	29.5	18.5
2	0.05	**15.5**	4.5	1.5	3.0	2.0	3.0	5.0	**26.5**	9.5	3.0	6.0	7.0	7.5	10.0	**44.0**	21.0	14.0	15.0	11.5	18.5	18.0
3	0.05	**13.0**	2.5	0.5	2.0	2.0	2.5	2.5	**27.5**	4.0	4.0	4.0	2.5	3.5	4.5	**45.5**	13.5	15.5	8.0	9.0	17.0	11.5
4	0.05	**14.0**	3.5	1.5	2.5	1.5	3.5	3.0	**27.0**	9.5	4.0	6.5	6.0	7.5	8.5	**39.5**	18.5	7.5	13.5	13.5	16.0	16.0
5	0.05	**14.5**	3.0	0.5	2.0	1.5	2.5	3.5	**31.0**	8.0	3.0	5.5	3.5	7.0	8.0	**47.0**	12.5	15.0	10.5	9.0	17.0	13.0
6	0.05	**15.0**	2.5	0.5	0.5	1.0	1.5	1.0	**30.0**	9.5	4.0	7.0	7.0	4.0	9.0	**45.5**	21.0	8.0	17.0	15.0	14.0	21.0
7	0.05	**18.0**	0.5	0.0	0.5	0.0	1.5	1.5	**29.5**	2.5	3.0	1.0	1.0	3.0	2.5	**44.0**	9.0	8.5	5.0	3.5	9.0	10.0
1	0.10	**14.5**	1.5	3.0	1.5	2.5	7.5	3.5	**30.5**	8.0	13.0	6.0	8.0	15.5	8.5	**48.5**	19.0	24.0	14.5	20.5	31.0	20.0
2	0.10	**9.5**	2.0	1.0	1.5	2.0	3.5	3.0	**24.5**	10.5	4.5	7.0	7.5	8.5	9.0	**40.0**	22.0	15.5	18.0	19.5	18.5	23.0
3	0.10	**11.5**	1.5	1.5	1.0	2.0	3.0	2.5	**27.0**	5.0	9.0	4.0	4.5	8.5	6.0	**41.5**	12.5	20.5	10.5	10.5	22.5	13.5
4	0.10	**11.0**	3.0	2.0	2.5	1.0	2.5	2.5	**20.5**	7.0	4.0	5.0	3.5	9.5	5.5	**39.5**	18.0	13.0	11.5	12.5	16.0	14.0
5	0.10	**13.5**	2.5	3.0	1.5	2.0	3.5	2.5	**24.0**	6.0	6.0	4.0	3.0	9.0	7.0	**40.5**	18.0	15.5	12.0	13.5	18.0	14.5
6	0.10	**6.5**	0.5	0.0	0.0	0.0	0.0	0.5	**17.0**	4.5	1.0	2.5	3.0	3.0	3.5	**36.0**	13.0	7.0	7.5	8.5	11.5	10.5
7	0.10	**7.5**	1.5	1.5	2.0	1.0	3.0	2.0	**22.5**	4.0	5.0	4.0	4.0	8.0	4.0	**40.5**	15.5	13.5	9.0	10.5	17.0	13.0
1	0.20	20.0	9.0	12.0	9.5	12.0	**22.5**	11.0	**41.0**	18.0	27.0	17.0	25.0	39.5	21.0	**59.0**	31.5	47.5	28.0	38.0	55.0	30.0
2	0.20	**8.5**	1.0	2.5	0.5	1.5	4.5	2.5	**22.0**	5.5	11.0	5.0	8.0	13.5	7.0	**45.0**	18.5	27.0	12.0	21.5	30.0	17.0
3	0.20	**9.0**	3.0	5.0	3.0	6.5	**9.0**	4.0	**25.5**	9.0	17.5	9.0	13.0	20.0	10.5	**44.5**	17.0	34.0	16.0	22.0	34.5	19.5
4	0.20	**8.5**	1.0	1.0	0.5	1.0	1.5	1.0	**21.5**	6.5	6.0	4.0	6.0	9.0	5.5	**38.0**	15.0	16.0	13.0	12.0	20.5	17.5
5	0.20	**12.5**	4.0	2.5	4.0	1.0	4.5	4.0	**29.0**	8.0	5.5	6.0	6.0	12.5	8.5	**43.5**	17.5	17.5	14.0	15.0	24.0	18.0
6	0.20	**5.5**	2.0	1.0	2.5	1.0	2.5	3.0	**21.5**	4.0	3.0	5.0	3.5	7.0	6.0	**35.0**	15.5	12.0	10.0	11.0	15.5	15.0
7	0.20	**8.5**	2.5	1.0	1.5	1.0	1.5	3.0	**21.0**	6.0	3.0	5.0	5.0	3.5	7.0	**42.0**	16.5	9.0	13.0	12.5	14.0	16.0

Method with greatest power emboldened.

* See text for description of genetic architecture for each scenario.

*RAML* Rare admixture maximum likelihood, *SKAT-O* sequence kernel association test, *T1* fixed threshold test 1 per cent MAF, *T5* fixed threshold test 5 per cent MAF, *WST* weighted sum test, *VTT* variable threshold test, *EREC* estimated regression coefficient test.

**Table 3:** Power (%) of seven methods to detect association of rare variants under seven scenarios for underlying genetic architecture using data simulated for TERT in 2000 cases and 2000 controls

		**Threshold for significance**																				
		**P < 0.001**							**P < 0.01**							**P < 0.05**						
Scenario*	**Proportion of variants associated**	**RAML**	**SKAT-O**	**T1**	**T5**	**WST**	**VTT**	**EREC**	**RAML**	**SKAT-O**	**T1**	**T5**	**WST**	**VTT**	**EREC**	**RAML**	**SKAT-O**	**T1**	**T5**	**WST**	**VTT**	**EREC**
1	0.05	**15.5**	10.5	3.5	4.0	4.0	6.5	10.5	**34.0**	22.5	14.0	16.0	14.5	15.0	20.5	**48.5**	36.0	25.5	22.5	24.0	30.0	34.0
2	0.05	**19.5**	13.0	5.0	4.0	3.5	7.0	13.5	**28.5**	21.5	11.5	11.0	12.0	14.5	19.0	**42.5**	38.5	24.0	21.5	24.0	25.0	36.5
3	0.05	**18.0**	10.5	6.5	4.5	3.5	5.5	6.5	**33.5**	17.5	12.5	7.0	11.0	12.0	18.5	**47.0**	35.5	26.0	18.0	25.0	25.5	33.5
4	0.05	**13.0**	5.0	2.0	1.0	1.0	1.0	3.0	**22.5**	13.5	7.0	4.0	5.0	6.0	9.0	**38.5**	27.5	14.0	14.5	14.5	12.5	25.5
5	0.05	**21.5**	13.5	4.5	4.5	3.0	6.0	9.0	**32.5**	21.0	10.0	8.5	9.0	14.0	17.5	**49.0**	35.5	19.5	20.0	20.0	20.5	36.5
6	0.05	**15.0**	8.5	3.0	4.0	2.0	5.0	6.5	**26.5**	13.5	7.5	7.0	7.0	10.0	12.0	**39.0**	25.0	16.5	12.5	13.5	15.5	22.0
7	0.05	**10.5**	4.5	0.0	2.0	1.5	2.5	4.5	**24.0**	10.5	3.0	7.5	4.0	4.5	10.0	**38.5**	25.0	11.0	13.0	10.5	16.0	22.5
1	0.10	**23.0**	18.5	12.5	15.0	13.0	16.0	19.0	**41.0**	32.0	26.0	24.5	27.5	31.0	34.0	**63.0**	51.0	43.5	39.5	48.0	44.0	53.5
2	0.10	**14.5**	9.0	7.0	3.5	5.0	7.0	7.5	**25.0**	20.5	15.5	8.0	12.0	13.0	19.5	**42.5**	36.0	23.5	23.5	24.0	25.0	34.0
3	0.10	**17.5**	11.5	6.5	6.5	7.0	8.5	10.5	**33.0**	23.5	19.5	14.5	18.5	22.0	22.0	**47.5**	39.0	33.0	27.0	33.5	32.5	39.5
4	0.10	**10.5**	10.0	4.5	5.5	4.5	5.0	7.5	**24.5**	18.0	11.0	11.0	10.5	12.0	16.5	**42.5**	32.5	20.0	21.0	20.5	22.5	34.0
5	0.10	**12.5**	11.0	3.5	6.0	5.5	5.5	10.0	**27.0**	20.0	10.5	14.0	11.0	13.0	22.5	**39.5**	35.5	28.0	27.0	23.5	26.0	34.5
6	0.10	**6.5**	6.0	3.0	0.0	2.0	2.5	4.5	**23.0**	14.0	7.0	6.5	6.0	7.5	12.5	**40.0**	27.5	17.0	13.5	17.0	18.5	26.0
7	0.10	**10.5**	7.0	2.0	0.5	2.0	2.0	6.0	**21.0**	15.0	5.5	7.5	7.0	6.5	12.5	**35.5**	25.5	13.5	14.0	13.5	16.0	24.0
1	0.20	31.0	**33.5**	31.5	24.5	30.5	30.5	30.5	55.0	58.0	53.0	48.5	**61.0**	55.5	58.5	75.0	72.0	73.5	67.5	**75.5**	70.5	73.0
2	0.20	**12.5**	12.0	9.5	6.5	9.5	10.0	**12.5**	**34.0**	30.5	25.0	20.0	26.0	26.0	29.5	**63.5**	53.5	45.5	37.0	45.0	42.0	55.0
3	0.20	**18.0**	14.5	11.0	11.0	14.5	15.5	16.0	**39.0**	36.5	32.0	23.5	33.0	31.5	35.0	**58.5**	**58.5**	49.5	45.0	49.5	45.5	56.5
4	0.20	**12.5**	10.5	5.5	3.0	3.0	4.5	7.5	**23.5**	18.5	16.0	16.0	16.0	17.5	20.0	**46.0**	40.5	31.5	28.5	31.5	31.0	39.0
5	0.20	**14.5**	10.5	6.5	4.5	5.0	6.5	10.5	**28.5**	27.0	20.0	17.5	19.0	17.5	26.5	**48.5**	47.0	36.5	30.0	32.5	36.0	43.0
6	0.20	**11.5**	6.0	2.0	3.0	1.0	2.0	4.0	**27.0**	15.5	7.0	7.5	8.0	8.0	17.5	**43.0**	38.0	19.5	21.0	20.0	21.5	33.5
7	0.20	**9.5**	4.0	1.0	2.0	1.0	1.0	4.5	**22.0**	17.5	5.5	5.5	6.0	5.5	13.0	**44.5**	30.0	15.0	13.0	11.5	17.5	28.5

Method with greatest power emboldened.

* See text for description of genetic architecture for each scenario.

*RAML* Rare admixture maximum likelihood, *SKAT-O* sequence kernel association test, *T1* fixed threshold test 1 per cent MAF, *T5* fixed threshold test 5 per cent MAF, *WST* weighted sum test, *VTT* variable threshold test, *EREC* estimated regression coefficient test.

Given the limited power of all the methods for the analysis of 2,000 cases and 2,000 controls, we repeated the evaluation of RAML and SKAT-O using data simulated for 4,000 cases and 4,000 controls across the same genomic regions. There were 145 rare variants (MAF < 0.04) in *BRCA1* (109 variants with MAF < 0.01, 27 with 0.01 < MAF < 0.02, 9 with 0.02 < MAF < 0.04), 274 rare variants in *BRCA2* (196, 22, 56) and 193 rare variants in *TERT* (155, 23, 15). The power of the two methods for 21 scenarios at the three thresholds for statistical significance are shown in Tables 4, 5 and 6 for *BRCA1*, *BRCA2* and *TERT* respectively. As expected, the power of the both methods is improved. The RAML method was still more powerful than SKAT-O under most scenarios, but the difference was greater than for the smaller sample sizes.

**Table 4:** Power (%) of RAML and SKAT methods to detect association of rare variants under seven scenarios for underlying genetic architecture using data simulated for BRCA1 in 4000 cases and 4000 controls

		**Threshold for significance**					
		**P < 0.001**		**P < 0.01**		**P < 0.05**	
Scenario*	**Proportion of variants associated**	**RAML**	**SKAT-O**	**RAML**	**SKAT-O**	**RAML**	**SKAT-O**
1	0.05	**47.5**	31.5	**61.0**	44.0	**73.5**	58.5
2	0.05	**39.5**	24.0	**53.5**	36.0	**64.5**	49.0
3	0.05	**43.0**	29.5	**61.5**	43.0	**70.5**	55.0
4	0.05	**33.5**	22.5	**49.5**	29.0	**59.5**	42.5
5	0.05	**39.5**	25.5	**57.0**	39.5	**62.0**	51.5
6	0.05	**33.5**	21.5	**43.0**	29.5	**57.0**	41.5
7	0.05	**37.5**	30.5	**51.0**	38.0	**56.5**	45.5
1	0.10	**40.0**	34.5	**58.0**	50.0	**76.0**	66.0
2	0.10	**36.0**	29.0	**50.5**	42.5	**65.5**	58.0
3	0.10	**32.5**	28.5	**53.0**	42.5	**67.5**	58.0
4	0.10	**31.0**	22.0	**45.5**	35.5	**59.0**	48.5
5	0.10	**33.5**	25.0	**55.0**	39.5	**71.0**	59.5
6	0.10	**32.5**	21.5	**48.5**	31.5	**62.0**	48.5
7	0.10	**31.5**	23.0	**44.0**	35.0	**60.5**	46.5
1	0.20	47.5	**50.0**	**71.0**	67.5	**83.5**	81.0
2	0.20	26.5	**34.5**	**49.0**	46.0	**69.5**	68.0
3	0.20	**41.0**	40.5	**59.5**	52.5	**74.0**	67.0
4	0.20	**26.0**	**26.0**	**44.5**	40.0	**67.5**	55.5
5	0.20	35.0	26.5	**51.5**	44.5	**70.0**	64.5
6	0.20	27.5	24.5	**45.5**	37.0	**62.5**	55.0
7	0.20	23.5	19.0	**46.0**	35.5	**63.0**	59.5

Method with greatest power emboldened.

* See text for description of genetic architecture for each scenario.

*RAML* Rare admixture maximum likelihood, *SKAT-O* sequence kernel association test, *T1* fixed threshold test 1 per cent MAF, *T5* fixed threshold test 5 per cent MAF, *WST* weighted sum test, *VTT* variable threshold test, *EREC* estimated regression coefficient test.

**Table 5:** Power (%) of RAML and SKAT methods to detect association of rare variants under seven scenarios for underlying genetic architecture using data simulated for BRCA2 in 4000 cases and 4000 controls

		**Threshold for significance**					
		**P < 0.001**		**P < 0.01**		**P < 0.05**	
Scenario*	**Proportion of variants associated**	**RAML**	**SKAT-O**	**RAML**	**SKAT-O**	**RAML**	**SKAT-O**
1	0.05	**78.5**	14	**87.5**	22	**96**	42.5
2	0.05	**69.5**	8.5	**84**	18	**93**	36
3	0.05	**67.5**	9.5	**76.5**	21	**85**	38
4	0.05	**61.5**	6	**72**	18.5	**83.5**	32.5
5	0.05	**68.5**	8.5	**78.5**	16	**87**	35.5
6	0.05	**61**	12.5	**75.5**	17.5	**82**	30.5
7	0.05	**65**	9	**79**	21.5	**86.5**	37.5
1	0.10	**75**	14.5	**86**	23	**90.5**	40.5
2	0.10	**61.5**	13.5	**81.5**	22	**92.5**	39.5
3	0.10	**59**	9.5	**76**	20	**89**	34
4	0.10	**58.5**	7	**72**	20.5	**84**	41
5	0.10	**65**	9	**82**	18.5	**89.5**	39
6	0.10	**61**	9	**79**	15.5	**86.5**	31
7	0.10	**66.5**	5	**83.5**	17	**91**	36
1	0.20	**83.5**	19.5	**95**	41	**99**	63.5
2	0.20	**63**	9.5	**77**	22.5	**93.5**	41
3	0.20	**66.5**	7	**85.5**	22.5	**94**	43.5
4	0.20	**53**	8.5	**72.5**	21	**87**	38.5
5	0.20	**70.5**	10	**86**	25.5	**93**	44
6	0.20	**59**	11	**79**	20.5	**92**	41.5
7	0.20	**61**	5.5	**79.5**	18.5	**89.5**	43.5

Method with greatest power emboldened.

* See text for description of genetic architecture for each scenario.

*RAML* Rare admixture maximum likelihood, *SKAT-O* sequence kernel association test, *T1* fixed threshold test 1 per cent MAF, *T5* fixed threshold test 5 per cent MAF, *WST* weighted sum test, *VTT* variable threshold test, *EREC* estimated regression coefficient test.

**Table 6:** Power (%) of RAML and SKAT methods to detect association of rare variants under seven scenarios for underlying genetic architecture using data simulated for TERT in 4000 cases and 4000 controls

		**Threshold for significance**					
		**P < 0.001**		**P < 0.01**		**P < 0.05**	
Scenario*	**Proportion of variants associated**	**RAML**	**SKAT-O**	**RAML**	**SKAT-O**	**RAML**	**SKAT-O**
1	0.05	**75.5**	53	**87**	66	**90**	80.5
2	0.05	**72.5**	42.5	**85**	66	**90**	78
3	0.05	**68**	42.5	**79**	57.5	**88.5**	73
4	0.05	**62.5**	35	**76**	50.5	**86**	64
5	0.05	**57**	36.5	**74.5**	50	**84.5**	61.5
6	0.05	**57.5**	35.5	**71**	52.5	**84.5**	67.5
7	0.05	**58**	34	**73.5**	48.5	**83.5**	64.5
1	0.10	**84**	62	**90**	79	**93.5**	89.5
2	0.10	**68**	47.5	**80.5**	66.5	**87.5**	77.5
3	0.10	**68.5**	50	**81**	65	**89.5**	78
4	0.10	**56**	37	**70.5**	53.5	**80**	68.5
5	0.10	**66.5**	42.5	**80.5**	59	**90.5**	78
6	0.10	**57.5**	38.5	**79.5**	53.5	**88**	70
7	0.10	**63**	41	**83.5**	61	**91.5**	73.5
1	0.20	**90.5**	87	**96.5**	96	**99.5**	98
2	0.20	**70.5**	57.5	**84**	75.5	**93.5**	85.5
3	0.20	**74.5**	65.5	**89**	79	**96.5**	89.5
4	0.20	**66.5**	54	**80**	72	**92**	84
5	0.20	**72.5**	58	**86**	72.5	**92**	87.5
6	0.20	**57.5**	41	**76**	61	**90.5**	80
7	0.20	**60**	41.5	**74**	59	**86.5**	76.5

Method with greatest power emboldened.

* See text for description of genetic architecture for each scenario.

*RAML* Rare admixture maximum likelihood, *SKAT-O* sequence kernel association test, *T1* fixed threshold test 1 per cent MAF, *T5* fixed threshold test 5 per cent MAF, *WST* weighted sum test, *VTT* variable threshold test, *EREC* estimated regression coefficient test.

The apparent difference between RAML and SKAT-O for the *BRCA1* and the other two regions is related to the fact that SKAT-O does not use a fixed threshold for the minor allele frequency, but uses a weighting function based on the minor allele frequency [7]. Thus the method is sensitive to the number of variants around the threshold MAF of interest. There were relatively fewer variants with a MAF just above 4 per cent in *BRCA1* than in the other two genes.

## Conclusion

We have described a new method for association testing of multiple rare variants that makes no assumptions about the proportion of variants that are associated with the phenotype of interest or the magnitude and direction of their effect. The method is flexible and can be applied to both dichotomous and quantitative traits and allows for the inclusion of covariates in the underlying regression model. We have compared the performance of RAML with six other similar methods using data simulated under 21 plausible scenarios for the underlying genetic model of association. Under most of these scenarios, RAML was found to have the greatest power, although SKAT-O performed better under some circumstances.

Genome-wide association studies have been very successful in identifying common alleles associated with many disease and physiological traits. However, these alleles explain a small fraction of the genetic component of the variance for most traits. It is very likely that rare variants will contribute to some of the so-called missing heritability. A systematic search for disease associated rare variants has been made possible by the availability of high-throughput, affordable sequencing technologies and the development of genotyping arrays that include hundreds of thousands of rare variants. Given that the underlying genetic model for association between rare genetic variants and disease related phenotypes is not known – effect allele frequency, effect size and proportion of associated variants - it is not possible to provide a definitive guide to the situations in which RAML should be preferred to SKAT-O or other methods. Until empirical evidence emerges for association of multiple rare variants across a genomic region it would seem reasonable to use multiple methods for burden testing including both RAML and SKAT-O.

## Appendix

The AML and RAML software are available from http://ccge.medschl.cam.ac.uk/software/.

## Competing interests

The authors declare that they have no competing interests.

## Authors’ contributions

JPT and PDPP conceived the idea for the method. JPT, QG and DFE developed the statistical methodology. JPT and QG programmed the simulation studies. All authors helped draft and edit the final manuscript.
